# Standardized, systemic phenotypic analysis reveals kidney dysfunction as main alteration of *Kctd1*^*I27N*^ mutant mice

**DOI:** 10.1186/s12929-017-0365-5

**Published:** 2017-08-17

**Authors:** Sudhir Kumar, Birgit Rathkolb, Sibylle Sabrautzki, Stefan Krebs, Elisabeth Kemter, Lore Becker, Johannes Beckers, Raffi Bekeredjian, Robert Brommage, Julia Calzada-Wack, Lillian Garrett, Sabine M. Hölter, Marion Horsch, Martin Klingenspor, Thomas Klopstock, Kristin Moreth, Frauke Neff, Jan Rozman, Helmut Fuchs, Valérie Gailus-Durner, Martin Hrabe de Angelis, Eckhard Wolf, Bernhard Aigner

**Affiliations:** 1 Chair for Molecular Animal Breeding and Biotechnology, and Laboratory for Functional Genome Analysis, Gene Center, LMU Munich, 81377 Munich, Germany; 2 German Mouse Clinic, Institute of Experimental Genetics, Helmholtz Zentrum München, German Research Center for Environmental Health, 85764 Neuherberg, Germany; 3 Member of German Center for Diabetes Research (DZD), 85764 Neuherberg, Germany; 4 Research Unit Comparative Medicine, Helmholtz Zentrum München, German Research Center for Environmental Health, 85764 Neuherberg, Germany; 5 Department of Neurology, Friedrich-Baur-Institute, University Hospital Munich, 80336 Munich, Germany; 6 Chair of Experimental Genetics, Center of Life and Food Sciences Weihenstephan, TU Munich, 85350 Freising-Weihenstephan, Germany; 7 Department of Medicine III, Division of Cardiology, University of Heidelberg, 69120 Heidelberg, Germany; 8 Institute of Pathology, Helmholtz Zentrum München, German Research Center for Environmental Health, 85764 Neuherberg, Germany; 9 Institute of Developmental Genetics, Helmholtz Zentrum München, German Research Center for Environmental Health, 85764 Neuherberg, Germany; 10 Molecular Nutritional Medicine, Else Kröner-Fresenius Center, TU Munich, 85350 Freising-Weihenstephan, Germany; 11 German Center for Vertigo and Balance Disorders, University Hospital Munich, 81377 Munich, Germany; 12 Munich Cluster for Systems Neurology (SyNergy), 80336 Munich, Germany; 13 German Center for Neurodegenerative Diseases (DZNE), 80336 Munich, Germany

**Keywords:** Animal model, Kctd1, SEN syndrome, Systematic phenotype analysis

## Abstract

**Background:**

Increased levels of blood plasma urea were used as phenotypic parameter for establishing novel mouse models for kidney diseases on the genetic background of C3H inbred mice in the phenotype-driven Munich ENU mouse mutagenesis project. The phenotypically dominant mutant line HST014 was established and further analyzed.

**Methods:**

Analysis of the causative mutation as well as the standardized, systemic phenotypic analysis of the mutant line was carried out.

**Results:**

The causative mutation was detected in the potassium channel tetramerization domain containing 1 (*Kctd1*) gene which leads to the amino acid exchange *Kctd1* ^*I27N*^ thereby affecting the functional BTB domain of the protein. This line is the first mouse model harboring a *Kctd1* mutation. *Kctd1* ^*I27N*^ homozygous mutant mice die perinatally. Standardized, systemic phenotypic analysis of *Kctd1* ^*I27N*^ heterozygous mutants was carried out in the German Mouse Clinic (GMC). Systematic morphological investigation of the external physical appearance did not detect the specific alterations that are described in *KCTD1* mutant human patients affected by the scalp-ear-nipple (SEN) syndrome. The main pathological phenotype of the *Kctd1* ^*I27N*^ heterozygous mutant mice consists of kidney dysfunction and secondary effects thereof, without gross additional primary alterations in the other phenotypic parameters analyzed. Genome-wide transcriptome profiling analysis at the age of 4 months revealed about 100 differentially expressed genes (DEGs) in kidneys of *Kctd1* ^*I27N*^ heterozygous mutants as compared to wild-type controls.

**Conclusions:**

In summary, the main alteration of the *Kctd1* ^*I27N*^ heterozygous mutants consists in kidney dysfunction. Additional analyses in 9–21 week-old heterozygous mutants revealed only few minor effects.

**Electronic supplementary material:**

The online version of this article (doi:10.1186/s12929-017-0365-5) contains supplementary material, which is available to authorized users.

## Background

Biomedical research with mice as animal models includes the search for and the analysis of alleles that predispose for or protect against specific diseases. A strategy for the search of novel disease-related alleles consists in the random chemical mutagenesis of a large number of animals followed by systematic screening for clinically relevant disease phenotypes. The alkylating agent *N*-ethyl-*N*-nitrosourea (ENU) is mutagenic for premeiotic spermatogonial stem cells and allows the production of a large number of randomly mutagenized offspring from treated males. ENU predominantly induces point mutations [1]. In the phenotype-driven Munich ENU mouse mutagenesis project using C3HeB/FeJ (C3H) inbred mice as genetic background, a standardized screening profile of clinical chemical blood plasma parameters was established for the analysis of offspring of mutagenized mice in order to detect phenotypic variants [2, 3]. Several mutant lines were established showing increased plasma urea levels as a parameter that is indicative of kidney diseases [4].

*Kctd1* is a member of the potassium channel tetramerization domain (Kctd) gene family. The N-termini of KCTD proteins and some voltage-gated K^+^ (Kv) channels are homologous [5, 6]. KCTD1 is not a membrane protein and is unlikely to be an ion channel protein [7]. The KCTD1 protein contains a N-terminal BTB (bric-a-brac, tram track, broad complex) domain which is also known as POZ (poxvirus and zinc finger) domain. The BTB domain is a highly conserved motif of about 100 amino acids mostly identified at the N-terminus of over 200 human proteins, including transcription factors, oncogenic proteins, and ion channel proteins [8]. BTB domains are protein–protein interaction modules that mediate both self-association and interaction with non-BTB partners [9]. BTB domain–containing proteins predominantly serve as transcriptional repressors and have been implicated in many developmental processes. Consequently, mutations of these proteins are linked with cancers and developmental disorders [10]. *Kctd* expression studies revealed high levels in fetal tissues and low levels in adults suggesting their role during development [11]. The KCTD1 protein is expressed in the mammary gland, kidney, brain, and ovary [10].

KCTD proteins participate in a wide variety of cellular functions including transcription regulation, cellular proliferation, apoptosis, cell morphology, ion channel assembly, and protein degradation through ubiquitination [12, 13] (and refs. Therein). Unlike other BTB family proteins, KCTD1 does not contain any other domains [10]. KCTD1 acts as a nuclear protein by inhibiting the transactivation of the transcription factor AP-2α (TFAP2A) and other TFAP2 family members via its BTB domain [7]. In addition, KCTD1 functions as an inhibitor of Wnt signaling pathway [14].

*KCTD1* encodes a 257 amino acid protein with a predicted molecular mass of 29.4 kDa. The amino acid sequence of KCTD1 is highly conserved with humans and mice having the identical amino acid sequence. In addition, a 265-amino-acid isoform is described in mice differing by an 8 amino acid insertion immediately following the start codon. Human and rat KCTD1 differ only by a single amino acid residue [10] (http://www.ensembl.org; http://www.uniprot.org). The MGI database (http://www.informatics.jax.org) harbouring knockout as well as mutant mouse alleles includes no information about published *Kctd1* mouse mutants (as of 03.03.2017).

Apart from the short isoform (257 aa in humans and mice, and 265 aa in mice) harbouring only the BTB domain, a long isoform of KCTD1 is described (861 aa in mice) (http://www.ensembl.org). The long isoform contains both a N-terminal DUF3504 domain and a C-terminal BTB domain. The putative function of DUF3504 is suggested to be DNA or protein binding [15].

In humans, heterozygous *KCTD1* missense mutations occur in the scalp-ear-nipple (SEN) syndrome (OMIM 181270) or Finlay-Marks syndrome, which is a rare autosomal dominant disorder. All mutations were found in the highly conserved BTB domain. The identified missense mutations cause a loss of function via a dominant-negative mechanism. The disease is characterized by cutis aplasia of the scalp, minor anomalies of the external ears, digits and nails, and malformations of the breast. The penetrance of the SEN syndrome appears to be high, but the disease shows a substantially variable phenotype within affected families [16, 17]. Less frequent clinical observations include renal and urinary tract malformations also leading to hypertension. In addition, there are reports of partial manifestations in isolated or familial cases including putative recessive inheritance [18, 19] (and refs therein).

In addition, *KCTD1* has been associated in genome-wide association studies (GWAS) with sudden cardiac arrest due to ventricular tachycardia or ventricular fibrillation in patients with coronary artery disease [20]. Tentative associations with blood pressure were also described for *KCTD1* [21].

The ENU mutagenesis-derived dominant mutant mouse line HST014 showing increased plasma urea levels was analyzed for the causative mutation. After the identification of the mutation in *Kctd1*, a standardized, systemic phenotypic analysis of *Kctd1* ^*I27N*^ heterozygous mutant mice was carried out in the German Mouse Clinic (GMC, http://www.mouseclinic.de) to examine organ systems and/or pathways that may be affected by the *Kctd1* mutation as primary or secondary effects. The results should give hints for the assessment of the mutant line as a model for the published heterozygous *KCTD1* mutations in humans.

## Methods

### Animals, linkage analysis, and detection of the causative mutation

The dominant mutant line HST014 was established in the clinical chemical screen of the phenotype-based Munich ENU mouse mutagenesis project [22] on the C3HeB/FeJ (C3H) inbred genetic background by detecting increased plasma urea values at the age of three months (cut-off level: 70 mg/dl, or 11.7 mmol/l). Mouse husbandry, breeding, linkage analysis, and genome-wide mapping were performed as described previously [4]. All mice had free access to drinking water and a standard rodent diet (V1124, Ssniff, Soest, Germany; Altromin chow #1314, Altromin, Lage, Germany) ad libitum.

For linkage analysis of the causative mutation, automated DNA extraction from tissue lysates was performed using the AGOWA Mag Maxi DNA Isolation Kit (AGOWA, Berlin, Germany). A genome-wide mapping panel consisting of single nucleotide polymorphism (SNP) markers was applied. The markers used are available upon request. Genotyping using this panel was performed by MassExtend, a MALDI-TOF high-throughput genotyping system supplied by Sequenom (San Diego, CA, USA). Additional fine mapping was performed using further SNP and microsatellite markers. Chromosomal positions of markers and genes are according to the GRCm38.p4 mouse genome assembly, 2016 (http://www.ensembl.org). All genes located within the identified defined chromosomal region were examined for published data about their wild-type and mutant function with respect to their potential impact on renal function and renal diseases.

### Exome sequencing

Genomic DNA from three controls and one mouse carrying the putative mutation was sheared by sonication (Bioruptor, Diagenode, Liege, Belgium), end-repaired, A-tailed and ligated to Illumina adapters. The resulting whole genome sequencing libraries were amplified by six cycles of PCR and then hybridized to a mouse whole exome bait library. Fragments complementary to the biotinylated exome bait library were enriched by pull-down with paramagnetic streptavidin-coated beads (Dynabeads M280, Invitrogen, USA) and finally amplified with barcoded Illumina adapters. All previously described steps used reagents from the Agilent whole exome kit and followed the protocol of the manufacturer. The resulting exome libraries were purified with Ampure XP beads (Beckman-Coulter, USA), quantified and assessed on the Bioanalyser (Bioanalyser 2100, Agilent, Santa Clara, USA). Pooled, barcoded libraries were sequenced on an Illumina Genome Analyzer IIx in paired-end mode with a read length of 80 bp in either direction. Sequence reads in fastq format were demultiplexed, adapter-clipped and quality filtered. After mapping to the mouse genome with BWA, SNPs were called using VARSCAN. Only SNPs that were exclusively called in the mutant mouse and not in any of the three controls were kept for further evaluation.

### Phenotypic analysis in the German Mouse Clinic

Maintenance of the dominant mutant mouse line *Kctd1* ^*I27N*^ involved repeated backcross to C3H wild-type mice for more than ten generations, leading to the subsequent loss of all non-causative ENU mutations not linked to the *Kctd1* mutation. The comprehensive phenotypic analysis was carried out in the German Mouse Clinic at the Helmholtz Zentrum München by using standardized examination protocols (http://www.mouseclinic.de). The analysis covers several hundred parameters in the fields of allergy, behavior, cardiovascular analysis, clinical chemistry, dysmorphology including bone and cartilage, energy metabolism, eye morphology and vision, immunology, molecular phenotyping, neurology, nociception, and pathology. The complete protocols of the examinations are described under http://www.mouseclinic.de [23–25].

*Kctd1* ^*I27N*^ heterozygous mutant mice and wild-type control littermates were analyzed between 9 and 21 weeks of age (see Additional file 1). Fifteen mice were analyzed per sex and genotype (unless otherwise stated in the text of the Results section). In addition, a renal function test using metabolic cages for single mice (Tecniplast, Hohenpeissenberg, Germany) was performed as described previously [23] in a second cohort of mice at an age of 28–32 weeks comprising 12 animals per sex and genotype. Mouse husbandry was done under a continuously controlled specific pathogen free (SPF) hygiene standard according to the FELASA recommendations [26] (http://www.felasa.eu). All tests were carried out under the approval of the responsible animal welfare authority (Regierung von Oberbayern).

Data are shown as mean ± standard deviation. If not otherwise stated, data were analyzed using R, a language and environment for statistical computing. Tests for genotype effects were made by using Wilcoxon rank sum test or linear models depending on the assumed distribution of the parameter and the questions addressed to the data. For categorical data, Fisher’s exact test was used. Statistically significant differences are indicated for *P* < 0.05, 0.01, and 0.001.

## Results

### Generation of line Kctd1^I27N^ and identification of the causative mutation

The ENU mutagenesis-derived, dominant mutant line HST014 with the G1 male founder ID 10295828 was established on the C3H inbred genetic background by showing increased plasma urea values at the age of 3 months (cut-off level: 70 mg/dl, or 11.7 mmol/l). Complete penetrance of the mutant phenotype was observed in offspring of matings of phenotypically heterozygous mutant mice to wild-type C3H mice as expected by the rules of Mendelian inheritance. Heterozygous mutants of both sexes were viable and fertile, and showed no grossly apparent phenotype compared to wild-type controls.

Genome-wide linkage analysis of the causative mutation was carried out with 45 phenotypically heterozygous mutant G2 animals derived from two consecutive backcross matings of phenotypically heterozygous mutants to BALB/c inbred mice. Using a set of 113 SNPs, the mutant phenotype was mapped to the proximal region of MMU18. Further fine mapping showed the highest χ2 values for the polymorphic markers D18Mit68 (21.4 Mb; χ2 value: 21.4) and rs29827614 (25.3 Mb; χ2 value: 21.3). The three candidate genes *Kctd1* (15.0 Mb), *Aqp4* (15.4 Mb) and *Mep1b* (21.1 Mb) were chosen for sequence analysis. cDNA analysis of *Aqp4* and *Mep1b* resulted in identical sequences in wild-type and phenotypically heterozygous mutant animals, which were also identical to published sequences (http://www.ensembl.org).

**Fig. 1:**
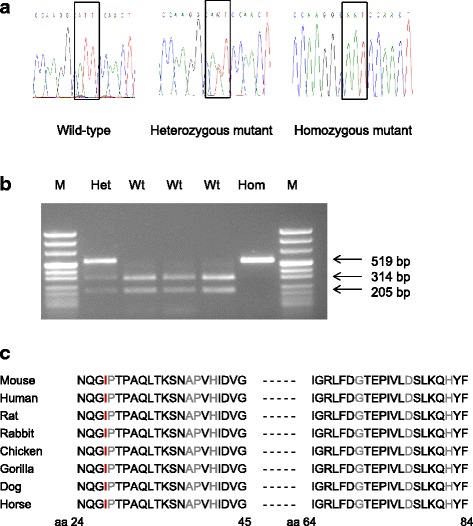
Analysis of *Kctd1* in wild-type and *Kctd1* ^*I27N*^ mutant mice. **a** Electropherogram of the *Kctd1* ^*I27N*^ mutation. The box shows the T→A transversion from the wild-type codon ATT (I) to the mutant codon AAT (N) at amino acid position 27 (265 aa isoform). **b** Genotyping of mice by allele-specific PCR-RFLP reaction. *Bsm*I restriction digest of the 519 bp PCR product results in 314 bp and 205 bp fragments of the wild-type allele. Hom, *Kctd1* ^*I27N*^ homozygous mutant; Het, *Kctd1* ^*I27N*^ heterozygous mutant; Wt, wild-type; M, pUC Mix 8 marker, MBI Fermentas. **c** Partial protein sequence alignment of murine KCTD1 with other species. Amino acid residue in red color shows the position of the *Kctd1* ^*I27N*^ mutation. Amino acid residues in grey color show the positions of mutations in humans affected by the SEN syndrome [17]. Amino acid number of the human sequence (257 aa) is murine amino acid number (265 aa isoform) minus 8

Sequence analysis of the *Kctd1* cDNA revealed a point mutation resulting in a T→A transversion at nucleotide 80 (ENSMUST00000025992.6, 265-amino-acid isoform), leading to an amino acid exchange from isoleucine to asparagine at position 27. Therefore, the name of line HST014 was designated as *Kctd1* ^*I27N*^. The mutation affects the highly conserved functional BTB domain of the protein. Allelic differentiation of the *Kctd1* ^*I27N*^ mutation was performed by PCR-RFLP since the point mutation abolished the restriction site for the enzyme *Bsm*I (Fig. 1). As the causative mutation mapped proximal to marker D18Mit68 at 21.4 Mb, calculation of the probability of the existence of confounding non-segregating mutations was done for the chromosomal region of 1 bp to 20 Mb of chromosome 18, and the probability turned out to be significantly low (*P* < 0.01) [27].

*Kctd1* ^*I27N*^ heterozygous mutants were crossed to generate homozygous mutant animals. Analysis of 47 offspring at 3 months of age without prior loss of animals after rearing from four mating pairs identified no homozygous mutants, but heterozygous mutants (*n* = 31; 66%) and wild-type mice (*n* = 16; 34%) in a 2:1 ratio. 21 fetuses at day E17.5 derived from three timed matings of heterozygous mutant mice appeared grossly normal in their morphology. Homozygous mutant (*n* = 5), heterozygous mutant (*n* = 10), and wild-type (*n* = 6) animals were observed according to the Mendelian ratio. Additional offspring (*n* = 70) of matings of heterozygous mutants were genotyped shortly after birth. The results confirmed that *Kctd1* ^*I27N*^ homozygous mutant mice showed perinatal death.

In addition, exome sequencing using genomic DNA of a heterozygous mutant mouse was carried out for the search for confounding mutations linked to the identified mutation *Kctd1* ^*I27N*^. In addition to the already detected point mutation in *Kctd1*, a second heterozygous point mutation was found in the gene *Dsg1b* (20.4 Mb on MMU 18) leading to the amino acid exchange *Dsg1b* ^*G993D*^ (ENSMUST00000076737.6) and being inherited together with the mutation *Kctd1* ^*I27N*^. Therefore, mutant offspring were selected carrying either only the mutation *Kctd1* ^*I27N*^ but not the second mutation *Dsg1b* ^*G993D*^ or vice versa. *Kctd1* ^*I27N*^ heterozygous mutant mice without the second mutation *Dsg1b* ^*G993D*^ also showed the primary phenotype of increased plasma urea levels, whereas *Dsg1b* ^*G993D*^ heterozygous mutant mice without the mutation *Kctd1* ^*I27N*^ showed inconspicuous plasma urea levels as well as the absence of another apparent pathological phenotype. Genotypic analysis of the progeny (*n* = 53) of *Kctd1* ^*I27N*^ heterozygous mutant mice without the second mutation *Dsg1b* ^*G993D*^ shortly after birth also confirmed that *Kctd1* ^*I27N*^ homozygous mutant mice showed perinatal death. The cause of the perinatal death of the homozygous mutant mice has to be carried out in future analyses.

### Phenotypic analysis in the German Mouse Clinic

Phenotypic analysis of mutant mice in the German Mouse Clinic includes the aim to collect and deliver by open access systemic phenome data of a high number of mutant mouse lines in a standardized manner. The complete phenotype reports of the *Kctd1* ^*I27N*^ mutant line described here as well as of the HST014 mutants carrying both mutations *Kctd1* ^*I27N*^ and *Dsg1b* ^*G993D*^ will be deposited online (http://tools.mouseclinic.de/phenomap/jsp/annotation/public/phenomap.jsf).

### Behavioral and neurological analysis

*Kctd1* ^*I27N*^ heterozygous mutant mice were tested at the age of 9–10 weeks by open field test, modified SHIRPA, grip strength, rotarod and acoustic startle response. Body weights of heterozygous mutant and wild-type mice showed no genotype-specific difference. The open field test showed minor effects for the number of rearings, the resting time and the latency to enter in the center (Table 1).

**Table 1:** Open field behavioral analysis of line *Kctd1* ^*I27N*^

Parameter	Heterozygous mutant males	Wild-type males	Heterozygous mutant females	Wild-type females	Genotype: *P*-value
Distance traveled – total (cm)	8834 ± 2939	10,050 ± 2329	9823 ± 2178	10,138 ± 2682	
Number of rearings - total	46 ± 41	75 ± 42	56 ± 45	74 ± 44	0.04
Percent distance in the center – total	10.4 ± 8.5	13.1 ± 7.3	12.5 ± 9.3	14.7 ± 8.3	
Percent time spent in the center - total	6.9 ± 6.1	9.0 ± 5.6	8.8 ± 7.9	10.2 ± 6.5	
Whole arena - resting time (sec)	47 ± 38	79 ± 36	70 ± 57	95 ± 58	0.03
Whole arena - average speed (cm/s)	7.7 ± 2.7	9.0 ± 2.3	8.8 ± 2.2	9.3 ± 2.8	
Latency to enter in the center (sec)	349 ± 377	125 ± 209	122 ± 228	81 ± 137	0.047
Number of entries in the center	59 ± 51	76 ± 45	77 ± 57	87 ± 54	

9-week-old mice were tested in the open field for 20 min. Number per genotype and sex: *n* = 15. Data are presented as mean ± standard deviation. Significance vs. wild-type controls: Exact *P* values are indicated for *P* < 0.05

The modified SHIRPA protocol is a semi-quantitative screening method for the overall qualitative analysis of abnormal phenotypes in mice using defined rating scales and includes 17 test parameters each contributing to the overall assessment of general health, posture and movement, autonomic reflexes, as well as behavioral aspects. *Kctd1* ^*I27N*^ heterozygous mutant mice showed similar locomotor activity (*P* = 0.4; no. of squares entered in the arena: 12.3 ± 9.0 vs. 14.4 ± 7.2 in males, and 9.7 ± 5.5 vs. 11.0 ± 8.2 in females) compared to wild-type controls. As phenotypical changes, they showed presence of tremor, presence of tail dragging, and presence of trunk curl (Table 2). All other SHIRPA parameters (body position, defecation and urination during observation, transfer arousal, gait, startle response, touch escape, limb grasping, pinna reflex, contact righting, evidence of biting, vocalization in and above the arena, unexpected behaviors, and head bobbing) were without significant alterations.

**Table 2:** Significant variations between mutant and wild-type mice in the modified SHIRPA and grip strength analysis of line *Kctd1* ^*I27N*^

Test	Parameter	Heterozygous mutant males	Wild-type males	Heterozygous mutant females	Wild-type females	Genotype: *P*-value
Modified SHIRPA	Presence of tremor	6 of 15	0 of 15	0 of 15	0 of 15	0.02
	Presence of tail dragging	12 of 15	4 of 15	6 of 15	2 of 15	0.003
	Presence of trunk curl	2 of 15	0 of 15	8 of 15	2 of 15	0.02
Grip strength	Four paws (g)	219 ± 9	260 ± 20	223 ± 18	236 ± 22	< 0.001

9-week-old mice were tested. Number per genotype and sex: *n* = 15. Data are presented as total numbers (modified SHIRPA) and mean ± standard deviation (grip strength). Significance vs. wild-type controls: Exact *P* values are indicated for *P* < 0.05 (Modified SHIRPA: Fisher’s exact test)

Measurement of the grip strength for two and four paws to evaluate muscle strength revealed reduced values in *Kctd1* ^*I27N*^ heterozygous mutants (Table 2). Evaluation of motor coordination and balance in three consecutive trials on the accelerating rotarod revealed no significant genotype-specific differences (latency to fall in sec for all three trials: *P* = 0.5; 113 ± 44 vs. 106 ± 37 in males, and 114 ± 48 vs. 105 ± 40 in females). The usual improvement in the performance of the task over the three trials as well as similar ratios of falling and passive rotation were observed in both genotypes.

There were no genotype-specific effects on prepulse inhibition of the acoustic startle response indicating that both sensorimotor gating and recruiting are intact in the mutants. Hearing sensitivity was assessed by measuring the auditory brainstem response (ABR) to different auditory stimuli in a subset of mice (*n* = 10 per sex and genotype), when the animals were 18 weeks old. There was no genotype-specific effect on hearing sensitivity.

In addition, analysis of the nociception by using the hotplate test at 12 weeks of age showed no differences between genotypes (data not shown).

### Cardiovascular analysis

*Kctd1* ^*I27N*^ heterozygous mutant mice were tested at the age of 15 weeks. Electrocardiography as well as evaluation of the left ventricular function by transthoracic echocardiography on conscious animals by analyzing the heart parameters interventricular septum in systole (IVSs), interventricular septum in diastole (IVSd), left ventricular posterior wall in systole (LVPWs), left ventricular posterior wall in diastole (LVPWd), left ventricular internal dimension in systole (LVIDs) and left ventricular internal dimension in diastole (LVIDd) were done. Only minor changes were found in *Kctd1* ^*I27N*^ heterozygous mutants, and none of the findings represents a pathological cardiovascular state (Table 3).

**Table 3:** Cardiovascular analysis of line *Kctd1* ^*I27N*^ by echocardiography

Parameter	Heterozygous mutant males	Wild-type males	Heterozygous mutant females	Wild-type females	Genotype: *P*-value
IVSs (mm)	0.55 ± 0.03	0.55 ± 0.02	0.55 ± 0.02	0.54 ± 0.02	
IVSd (mm)	0.54 ± 0.02	0.54 ± 0.02	0.55 ± 0.02	0.54 ± 0.02	0.04
LVPWs (mm)	0.54 ± 0.03	0.54 ± 0.02	0.54 ± 0.02	0.54 ± 0.02	
LVPWd (mm)	0.54 ± 0.03	0.56 ± 0.02	0.54 ± 0.01	0.55 ± 0.02	0.03
LVIDs (mm)	1.39 ± 0.49	1.44 ± 0.60	1.53 ± 0.56	1.69 ± 0.46	
LVIDd (mm)	2.87 ± 0.43	2.87 ± 0.57	2.85 ± 0.47	2.98 ± 0.32	
Heart rate (bpm)	579 ± 114	530 ± 150	474 ± 167	453 ± 122	
Respiration rate (1/min)	264 ± 36	250 ± 29	224 ± 50	238 ± 52	
Body weight (g)	31.3 ± 1.4	32.3 ± 1.9	26.8 ± 1.6	27.1 ± 2.1	

15-week-old mice were tested. Number per genotype and sex: *n* = 14–15. Data are presented as mean ± standard deviation. Significance vs. wild-type controls: Exact *P* values are indicated for *P* < 0.05

*IVSs* interventricular septum in systole, *IVSd* interventricular septum in diastole, *LVPWs* left ventricular posterior wall in systole, *LVPWd* left ventricular posterior wall in diastole, *LVIDs* left ventricular internal dimension in systole, *LVIDd* left ventricular internal dimension in diastole

### Clinical chemistry and hematology analysis

Line *Kctd1* ^*I27N*^ was established based on increased plasma urea levels in heterozygous mutant animals. Plasma urea was found to be significantly increased already at 6 and 9 weeks of age in both sexes (data not shown). Clinical chemical analysis of blood plasma at the age of 17 weeks revealed significantly increased levels of urea, creatinine, potassium, calcium, α-amylase activity and albumin in the heterozygous mutants. In addition, decreased levels for chloride and alkaline phosphatase activity were found in the heterozygous mutants (Table 4).

**Table 4:** Clinical chemical analysis of line *Kctd1* ^*I27N*^

Parameter	Heterozygous mutant males	Wild-type males	Heterozygous mutant females	Wild-type females	Genotype: *P*-value
Na (mmol/l)	157 ± 2	156 ± 2	156 ± 3	156 ± 2	
K (mmol/l)	5.1 ± 0.3	4.6 ± 0.2	4.4 ± 0.2	4.1 ± 0.2	< 0.001
Ca (mmol/l)	2.5 ± 0.1	2.5 ± 0.1	2.6 ± 0.1	2.5 ± 0.1	< 0.001
Cl (mmol/l)	113.5 ± 1.7	114.8 ± 1.7	113.9 ± 1.5	117.1 ± 1.4	< 0.001
P_i_ (mmol/l)	2.2 ± 0.2	2.2 ± 0.3	2.1 ± 0.3	2.1 ± 0.3	
Total protein (g/l)	51.3 ± 2.4	51.6 ± 1.9	50.7 ± 1.9	48.7 ± 1.6	
Creatinine (μmol/l)	9.4 ± 1.5	7.8 ± 1.2	10.6 ± 0.7	9.5 ± 1.2	< 0.001
Urea (mmol/l)	18.2 ± 1.5	10.8 ± 0.8	16.9 ± 1.4	10.1 ± 1.1	< 0.001
Cholesterol (mmol/l)	4.1 ± 0.4	4.2 ± 0.4	3.0 ± 0.3	2.8 ± 0.3	
Triglycerides (mmol/l)	4.1 ± 0.5	4.2 ± 1.2	3.4 ± 0.8	3.4 ± 1.0	
ALT (U/l)	37 ± 4	39 ± 9	32 ± 3	32 ± 3	
AST (U/l)	50 ± 6	54 ± 17	51 ± 7	51 ± 7	
AP (U/l)	90 ± 5	97 ± 8	132 ± 12	135 ± 8	0.04
α-Amylase (U/l)	776 ± 162	652 ± 54.	695 ± 91	534 ± 39	< 0.001
Glucose (mmol/l)	10.5 ± 2.0	9.2 ± 1.6	10.2 ± 1.5	9.8 ± 1.2	*P* > 0.05 (0.06)
Albumin (g/l)	28.7 ± 1.3	28.3 ± 1.3	30.8 ± 1.3	29.4 ± 1	0.02

17-week-old mice were tested. Number per genotype and sex: *n* = 11–14. Data are presented as mean ± standard deviation. Significance vs. wild-type controls: Exact *P* values are indicated for *P* < 0.05

*Creatinine* plasma creatinine analyzed by the enzymatic method, *ALT* alanine aminotransferase, *AST* aspartate aminotransferase, *AP* alkaline phosphatase

Hematological analysis of 17-week-old mice indicated decreased values for red blood cell count, hemoglobin, hematocrit, mean corpuscular volume and mean corpuscular hemoglobin in heterozygous mutant mice (Table 5). The erythropenic anemia may be due to kidney dysfunction. This alteration was also observed in other mutant mouse lines showing increased plasma urea levels which were established in the Munich ENU mouse mutagenesis project [28, 29]. Future measurement of plasma erythropoietin (EPO) levels may further clarify this point.

**Table 5:** Hematological analysis of line *Kctd1* ^*I27N*^

Parameter	Heterozygous mutant males	Wild-type males	Heterozygous mutant females	Wild-type males	Genotype: *P*-value
WBC (10^3^/μl)	10.6 ± 1.5	10.6 ± 2.0	10.9 ± 1.8	9.6 ± 1.2	
RBC (10^6^/μl)	9.5 ± 0.4	10.2 ± 0.3	9.8 ± 0.4	9.8 ± 0.3	0.001
PLT (10^3^/μl)	989 ± 158	1051 ± 72	982 ± 63	1002 ± 60	
HGB (g/dl)	15.4 ± 0.6	16.5 ± 0.5	15.7 ± 0.6	16.3 ± 0.8	< 0.001
HCT (%)	44.8 ± 1.7	48.1 ± 1.3	46.0 ± 1.7	46.6 ± 1.3	< 0.001
MCV (fl)	47.1 ± 0.6	47.3 ± 0.5	47.1 ± 0.5	47.6 ± 0.6	0.005
MCH (pg)	16.1 ± 0.5	16.2 ± 0.3	16.1 ± 0.3	16.6 ± 0.5	0.005
MCHC (g/dl)	34.3 ± 1.0	34.2 ± 0.5	34.3 ± 0.7	34.9 ± 1.0	

17-week-old mice were tested. Number per genotype and sex: *n* = 14–15. Data are presented as mean ± standard deviation. Significance vs. wild-type controls: Exact *P* values are indicated for *P* < 0.05

*WBC* white blood cell count, *RBC* red blood cell count, *PLT* platelet count, *HGB* hemoglobin, *HCT* hematocrit, *MCV* mean corpuscular volume, *MCH* mean corpuscular hemoglobin, *MCHC* mean corpuscular hemoglobin concentration

A fasting intraperitoneal glucose tolerance test (IpGTT) was carried out at the age of 14 weeks (*n* = 14–15 per genotype and sex). Heterozygous mutants showed slightly elevated basal glucose levels compared to wild-type controls (*P* = 0.06; 5.3 ± 0.4 mmol/l vs. 5.1 ± 0.6 mmol/l in males, and 4.8 ± 0.3 mmol/l vs. 4.6 ± 0.4 mmol/l in females), but no significant changes in the area under the curve for 120 min. Slightly elevated glucose levels of heterozygous mutants were also found in fed mice.

Renal function analysis using metabolic cages was carried out with additional animals at the age of 28–32 weeks. Water consumption was not significantly different between genotypes, but urinary volume was slightly increased and urine pH and osmolality were clearly altered in heterozygous mutant mice. Daily urine calcium and magnesium excretion were significantly increased in heterozygous mutant animals, while uric acid and albumin excretion were decreased compared to controls (Table 6).

**Table 6:** Urine analysis of line *Kctd1* ^*I27N*^

Parameter	Heterozygous mutant males	Wild-type males	Heterozygous mutant females	Wild-type females	Genotype: *P*-value
Body weight (g)	36.0 ± 1.9	37.1 ± 1.5	36.4 ± 3.3	37.5 ± 3.9	
Water ad libitum					
Water intake (ml/day)	3.8 ± 0.8	3.9 ± 0.8	4.1 ± 0.8	3.7 ± 0.5	
Urine volume (ml/day)	1.2 ± 0.2	1.0 ± 0.4	1.0 ± 0.3	0.8 ± 0.3	0.008
Osmolality (osmol/l)	2.3 ± 0.4	3.0 ± 0.7	2.9 ± 0.6	4.0 ± 0.9	< 0.001
Urine pH	5.9 ± 0.2	6.2 ± 0.3	6.1 ± 0.4	6.7 ± 0.3	< 0.001
Na (μmol/day)	103 ± 32	94 ± 23	99 ± 35	111 ± 25	
K (μmol/day)	371 ± 109	314 ± 141	384 ± 125	375 ± 92	
Ca (μmol/day)	4.6 ± 1.4	1.4 ± 0.6	7.2 ± 2.9	1.8 ± 0.4	< 0.001
Cl (μmol/day)	137 ± 46	139 ± 42	139 ± 47	175 ± 41	
Mg (μmol/day)	30 ± 9	19 ± 12	38 ± 12	31 ± 7	0.009
P_i_ (μmol/day)	264 ± 34	277 ± 123	257 ± 54	237 ± 46	
Creatinine (μmol/day)	4.7 ± 0.6	4.9 ± 1.8	4.3 ± 0.7	4.2 ± 0.5	
Urea (mmol/day)	1.6 ± 0.4	1.6 ± 0.6	1.7 ± 0.5	1.8 ± 0.4	
Uric acid (nmol/day)	318 ± 109	639 ± 205	490 ± 147	864 ± 203	< 0.001
Glucose (μmol/day)	2.1 ± 0.4	2.0 ± 0.9	2.3 ± 0.7	2.6 ± 0.4	
Total protein (mg/day)	13 ± 3	13 ± 5	2.5 ± 1.5	2.6 ± 1.9	
Albumin (μg/day)	115 ± 26	210 ± 93	86 ± 20	142 ± 53	< 0.001

28–32-week-old mice were tested for 48 h in metabolic cages. Number per genotype and sex: *n* = 12. Data are presented as mean ± standard deviation. Significance vs. wild-type controls: Exact *P* values are indicated for *P* < 0.05 (2-way ANOVA)

*Creatinine* urine creatinine analyzed by the enzymatic method

### Dysmorphology, bone and cartilage

*Kctd1* ^*I27N*^ heterozygous mutant mice were tested at 12 and 18 weeks of age. The systematic morphological investigation via visual inspection (shape, size, appearance of head, limbs, digits, tail; appearance of vibrissae, teeth, lips, genitalia; coat color, texture and appearance at multiple body sites; skin color, texture and appearance at multiple body sites) found no genotype-specific differences. X-ray analysis of the skeleton (skull, mandible, maxilla, teeth, orbital cavity, spine, ribs, scapula, clavicle, pelvis, femur, tibia, fibula, humerus, ulna, radius, digits, joints) also found no genotype-specific differences between heterozygous mutants and wild-type controls.

### Energy metabolism

Analysis of energy metabolism was done on 13-week-old mice under ad libitum feeding conditions. No major genotype-related differences were obvious during energy turnover measurements. The heterozygous mutants showed slight hypophagia and consequently slightly lower respiratory exchange ratios. Determination of body composition (fat mass, lean mass) by time domain nuclear magnetic resonance (TD-NMR) also showed similar results for heterozygous mutants and wild-type controls (Table 7) which was confirmed in the second measurement at 19 weeks of age (data not shown).

**Table 7:** Analysis of energy metabolism in line *Kctd1* ^*I27N*^

Test	Parameter	Heterozygous mutant males	Wild-type males	Heterozygous mutant females	Wild-type females	Geno-type: *P*-value
Indirect calori-metry	Body weight (g)	28.3 ± 1.2	29.2 ± 1.7	24.1 ± 1.6	24.0 ± 2.7	
	Food intake (g)	0.9 ± 0.5	1.2 ± 0.6	1.4 ± 0.8	1.7 ± 0.7	
	Average VO_2_ consumption (ml/h)	86 ± 6	88 ± 8	82 ± 10	82 ± 13	
	Average RER (VCO_2_/VO_2_)	0.79 ± 0.03	0.81 ± 0.02	0.81 ± 0.04	0.83 ± 0.04	0.03
	Average distance (cm in 20 min)	2777 ± 810	2386 ± 755	4467 ± 5870	3681 ± 3512	
TD-NMR	Body weight (g)	27.4 ± 1.2	28.4 ± 1.7	23.8 ± 1.4	24.1 ± 1.8	
	Fat mass (g)	5.7 ± 0.7	6.2 ± 0.9	5.5 ± 0.7	5.6 ± 0.9	
	Lean mass (g)	17.7 ± 0.6	18.1 ± 0.9	14.7 ± 0.8	14.9 ± 0.8	

Analysis was done on 13-week-old mice under ad libitum conditions. *n* = 14–15 per genotype and sex. Data are presented as mean ± standard deviation. Significance vs. wild-type controls: Exact *P* values are indicated for *P* < 0.05

*RER* respiratory exchange ratio, *TD-NMR* time domain nuclear magnetic resonance

### Pathology

Analysis in the phenotype areas of allergy, eye as well as immunology also showed no genotype-specific differences in *Kctd1* ^*I27N*^ heterozygous mutants (data not shown).

The pathological analysis was carried out in 21-week-old animals (*n* = 5 per sex and genotype). Macroscopic examination of the organs (adrenal gland, blood vessels, body weight, bone, brain, cartilage, cerebellum, cervix, colon, duodenum, epididymis, esophagus, eyes, funiculus spermaticus, heart, jejunum, kidneys, liver, lung, lymph nodes, male accessory sex glands, mammary gland, ovaries, pancreas, parathyroid, pituitary gland, prostate, rectum, salivary glands, skeletal muscle, skin, spinal cord, spleen, stomach, teeth, testis, thymus, thyroid, tongue, trachea, urinary bladder, uterus, vagina) and histological analysis of the above mentioned organs including the kidneys (HE staining) showed no genotype-specific differences.

### Additional phenotypic analysis of heterozygous mutants harboring both mutations Kctd1^I27N^ and Dsg1b^G993D^

In addition to the results of the phenotypic analysis of the *Kctd1* ^*I27N*^ heterozygous mutants described above, heterozygous mutants harboring both mutations *Kctd1* ^*I27N*^ and *Dsg1b* ^*G993D*^ were previously analyzed in the German Mouse Clinic before the successful segregation of the mutation *Dsg1b* ^*G993D*^ was carried out.

Mice homozygous for a targeted mutation in *Dsg1b* are described to exhibit increased bone mineral content as well as abnormal eye morphology, whereas humans with various heterozygous mutations in the human ortholog *DSG1* are described to show palmoplantar keratoderma I (OMIM: 148700) and erythroderma with palmoplantar keratoderma, hypotrichosis, and hyper-IgE (OMIM: 615508) (http://www.informatics.jax.org).

Heterozygous mutants harboring both mutations *Kctd1* ^*I27N*^ and *Dsg1b* ^*G993D*^ and wild-type control littermates were analyzed at an age of 2–4 months. The results (data not shown) essentially confirm the phenotype of the *Kctd1* ^*I27N*^ heterozygous mutants described above. Thus, in the behavioral and neurological analysis, open field test, grip strength, rotarod and hotplate test did not reveal genotype-specific differences. In the modified SHIRPA protocol, the mutants showed significantly decreased locomotor activity (*P* < 0.01) and also less tail elevation (*P* < 0.001). The significantly increased prepulse inhibition of the mutants carrying both mutations was not reproduced in the analysis of the *Kctd1* ^*I27N*^ heterozygous mutants described above. The clinical-chemical analysis confirmed the changes concerning plasma urea, potassium, chloride and α-amylase activity levels as well as the alterations of hematological parameters. Metabolic cage analysis at the age of 14–15 weeks found an increase in water intake and polyuria as well as distinct hypercalciuria in the mutants. Morphological analysis via visual inspection and x-ray and DXA (dual energy X-ray absorption) analysis (determining bone mineral density (BMD), bone mineral content (BMC), bone content, lean mass and fat mass at the age of 17 weeks p.p.) as well as the pathological analysis (macroscopic analysis of the organs and histological analysis) found no genotype-specific differences. Thus, unlike in other ENU-derived mutant mice showing hypercalciuria as consequence of kidney dysfunction [29–32], heterozygous mutants harboring both mutations *Kctd1* ^*I27N*^ and *Dsg1b* ^*G993D*^ showed no significant alterations in the parameters of bone mineralization at the analyzed age. This may be re-analyzed in older *Kctd1* ^*I27N*^ heterozygous mutants. In addition, absence of pathological states was described in the cardiovascular analysis and for the energy metabolism as well as in the analyses of allergy, eye, immunology, and lung function (data not shown).

Transcriptome profiling was carried out in heterozygous mutants harboring both mutations *Kctd1* ^*I27N*^ and *Dsg1b* ^*G993D*^. Based on the basal phenotype of the mutant line as well as expression and GWAS analyses in humans [10, 20], the pathogenic effects of the *Ktcd1* mutation were evaluated in brain, heart and kidney by genome-wide transcriptome profiling analysis using Illumina MouseRef8 v2.0 Expression Bead Chips containing about 25 K probes (25,600 well-annotated Ref-Seq transcripts). Samples of selected organs (brain, heart and kidney) of four mutant males were compared to four wild-type males as controls at the age of 4 months (in total 24 hybridizations). The raw data are available in the GEO database (http://www.ncbi.nlm.nih.gov/geo/). Statistical analysis of the gene expression patterns [33, 34] in the brain identified 25 significantly regulated genes in mutants vs. wild-type controls (false discovery rate (FDR) = 2.7%, fold change >1.5). The range of the mean log_2_ ratios was 1.4 – 2.2 for the 17 up-regulated genes and 2.8 to−1.5 for the 8 down-regulated genes. Statistical analysis of gene expression patterns in the heart identified 23 significantly regulated genes (FDR = 1.6%, fold change >1.5). The range of the mean log_2_ ratios was 1.4 – 1.7 for the 9 up-regulated genes and 1.8−1.4 for the 14 down-regulated genes. Due to the low number of regulated genes in brain and heart the functional classification of these datasets revealed no over-representation in gene ontology (GO) terms. Additionally, a detailed literature-based research for the genes regulated in brain and heart also indicated no clear functional changes on molecular level in these organs. Statistical analysis of the gene expression patterns in the kidney identified 102 significantly regulated genes in mutants vs. wild-type controls (FDR = 3.8%, fold change >1.5). The range of the mean log_2_ ratios was 1.4 – 2.5 for the 61 up-regulated genes and 2.9−1.4 for the 41 down-regulated genes. Using Ingenutity Pathway Analysis (IPA, http://www.ingenuity.com) the regulated genes in kidney were classified by their molecular functions and over-represented GO terms were associated with tumorigenesis, cell death, cell movement, hypertension, non-insulin-dependent diabetes mellitus, inflammatory bowel disease, as well as renal and urological disorder. The data need to be functionally verified in further experiments by using mice harboring the mutation *Kctd1* ^*I27N*^ without the second mutation *Dsg1b* ^*G993D*^.

## Discussion

The dominant mutant mouse line HST014 was established on the C3H inbred genetic background based on increased plasma urea levels, and the causative mutation was identified as *Kctd1* ^*I27N*^ affecting the highly conserved BTB domain of the protein. *Kctd1* ^*I27N*^ heterozygous mutant animals were viable and fertile whereas homozygous mutant animals showed perinatal death.

The data support the role of KCTD1 in mammalian development. *Kctd1* was shown to inhibit the transactivation of the *Tfap2* transcription factor family which plays an important role in embryogenesis and development. Homozygous knockout mice for *Tfap2a*, *Tfap2b* or *Tfap2c* exhibit lethal phenotypic defects in neural tube, face, eye, heart, skin, urogenital tissues or extraembryonic trophoblasts and usually die at birth [7]. In addition, *Tfap2b* homozygous knockout mice show neonatal or postnatal lethality with renal kidney cysts, depending on the genetic background [35, 36].

The transcriptional repressor activity of KCTD1 is mediated via the BTB domain [7]. In vitro mutagenesis of critical amino acid residues in the BTB domain resulted in the loss of BTB-dependent repression [37]. In humans affected by the SEN syndrome, all heterozygous KCTD1 missense mutations were identified in the BTB domain. The *Kctd1* ^*I27N*^ mutation also alters the BTB domain of the murine KCTD1 and presumably causes a loss of function via a dominant-negative mechanism.

The human SEN syndrome is characterized by cutis aplasia of the scalp as well as minor anomalies of the external ears, digits and nails, and malformations of the breast. The phenotype varies within the affected families. Less frequent clinical characteristics include renal and urinary tract malformations [17, 19]. In addition, *KCTD1* has been associated with sudden cardiac arrest [20]. A possible role of *KCTD1* in the brain was shown by its interaction with PrP^C^ in vitro [38].

Nine-to-21 week-old *Kctd1* ^*I27N*^ heterozygous mutant mice were examined using a standardized, systemic phenotypic analysis. Assessment of the reproducibility of the results was done by using the data of the preceding phenotypic analysis of heterozygous mutants harboring both mutations *Kctd1* ^*I27N*^ and *Dsg1b* ^*G993D*^. The concise analysis of dysmorphology, bone and cartilage as well as the pathological analysis revealed no visible alterations of coat, external ears, digits and nails as well as nipples characteristic for the SEN syndrome in humans. Further analysis of the mutation in other mouse inbred strains might examine the role of the genetic background on the detected mutant phenotype. The main alteration of *Kctd1* ^*I27N*^ heterozygous mutants in C3H mice consists in kidney dysfunction. We found no gross histological alternation in the kidneys of the mutant mice. This was also observed in other mutant mouse lines showing increased plasma urea levels which were established in the Munich ENU mouse mutagenesis project [30, 32]. Clinical chemical blood analysis and metabolic cage analysis of 17-week old and 28–32-week old heterozygous mutant mice, respectively, revealed signs of impaired kidney function including increased plasma urea, creatinine, and potassium levels, a slight increase in water intake, mild polyuria, and hypercalciuria. However, up to now creatinine clearance (calculated according to the formula: creatinine clearance = [Crea]_24-h urine_ × 24-h urine volume / [Crea]_plasma_; data were normalized to 25 g body weight) as an indicator for the general excretion function of the kidney showed no lower values in mutants compared to wild-type controls (data not shown). The specific cause of the observed functional renal alteration is not yet known and is a topic of subsequent analyses.

The altered RNA expression patterns in the kidneys of heterozygous mutants confirmed the clinical results. The data support the role of KCTD1 in renal development and/or physiology. These actions might partly be mediated by *Tfap2a*, since several genes that were found to be regulated in kidneys of *Kctd1* ^*I27N*^ heterozygous mutants have been identified as potential target genes of *Tfap2a* [39]. In this context, the findings in homozygous knockout mice of *Tfap2b* on the congenic 129P2 genetic background also included symptoms of kidney dysfunction like defective tubular secretory function and ion homeostasis, hypocalcemia, phosphatemia, hyperuremia, and terminal renal failure [35]. For the SEN syndrome in humans, renal anomalies were suggested to be more common manifestations of the disease than anticipated. Renal defects might contribute to morbidity and mortality via renal insufficiency and renovascular hypertension. Thus, all patients with SEN syndrome should undergo detailed renal evaluation [18].

The other analyses revealed few minor effects in cardiovascular and neurological parameters, and the results of the *Kctd1* ^*I27N*^ heterozygous mutants were within the physiological range of wild-type C3H mice. Analysis of RNA expression patterns also revealed few genes that were regulated in heart and brain, and did not indicate any clear tendency of alterations in these organs.

## Conclusions

In summary, *Kctd1* ^*I27N*^ homozygous mutant mice on the C3H inbred genetic background show perinatal death. As the first mouse line harbouring a *Kctd1* mutation, phenotypic analysis of *Kctd1* ^*I27N*^ heterozygous mutants at the age of 9–21 weeks revealed no visible alterations of the external physical appearance as observed in the human SEN syndrome. The main alteration of the *Kctd1* ^*I27N*^ heterozygous mutants consists in kidney dysfunction. Additional analyses in 9–21 week-old heterozygous mutants revealed only few minor effects.

## Additional file

Additional file 1: Time points of the phenotypic analyses for line *Kctd1* ^*I27N*^ in the German Mouse Clinic (GMC) (DOCX 32 kb)
