# Supplementary material for: Standardized, systemic phenotypic analysis reveals kidney dysfunction as main alteration of Kctd1I27N mutant mice
Source: J Biomed Sci. 2017 Aug 17;24:57. doi: 10.1186/s12929-017-0365-5 (PMC5559776; doi:10.1186/s12929-017-0365-5)
Supplement: Additional file 1: — Time points of the phenotypic analyses for line Kctd1 I27N in the German Mouse Clinic (GMC) (DOCX 32 kb) [file 12929_2017_365_MOESM1_ESM.docx]

**Additional file 1. Time points of the phenotypic analyses for line *Kctd1^I27N^* in the German Mouse Clinic (GMC)**

| Phenotypic analysis |  | Age in weeks |
| --- | --- | --- |
| Start and end of the GMC analysis |  | 9-21 |
| Behavior | Open field | 9 |
| Neurology | Modified SHIRPA, grip strength, rotarod, acoustic startle and prepulse inhibition | 9-10 |
| Nociception | Hotplate | 12 |
| Dysmorphology | Morphological observation | 12 |
| Metabolism | Indirect calorimetry, time domain nuclear magnetic resonance | 13 and 19 |
| Clinical chemistry | Simplified intraperitoneal glucose tolerance test | 14 |
| Cardiovascular | Electrocardiography, echocardiography | 15 |
| Eyes | Scheimpflug analysis, optical coherence tomography, eye size | 16 (no obvious differences; not shown) |
| Clinical chemistry | Clinical chemistry, hematology | 11 and 17 |
| Immunology | FACS analysis | 17 (no obvious differences; not shown) |
| Allergy | Plasma IgE level | 17 (no obvious differences; not shown) |
| Dysmorphology | X-ray | 18 |
| Neurology | Auditory brain stem response | 18 |
| Pathology | Macroscopical and histological analysis | 21 |

The GMC analyses were carried out in heterozygous mutants and homozygous wild-type mice as controls.
